# Echocardiographic evaluation of left ventricular function using an automated analysis algorithm is feasible for beginners and experts: comparison with invasive and non-invasive methods

**DOI:** 10.1007/s12574-022-00590-9

**Published:** 2022-10-13

**Authors:** Philipp Nicol, Andreas Rank, Tobias Lenz, Friederike Schürmann, Finn Syryca, Teresa Trenkwalder, Wibke Reinhard, Rafael Adolf, Martin Hadamitzky, Adnan Kastrati, Michael Joner, Heribert Schunkert, Leif-Christopher Engel

**Affiliations:** 1 Klinik Für Herz- und Kreislauferkrankungen, Deutsches Herzzentrum München, Technische Universität München, Munich, Germany; 2 Institut Für Radiologie und Nuklearmedizin, Deutsches Herzzentrum München, Technische Universität München, Munich, Germany; 3 Deutsches Zentrum Für Herz- und Kreislauf-Forschung (DZHK) E.V. (German Center for Cardiovascular Research), Partner Site Munich Heart Alliance, Munich, Germany

**Keywords:** Echocardiography, Imaging, Heart failure, Magnetic resonance imaging

## Abstract

**Aims:**

Echocardiographic measurement of left ventricular function using a user-friendly automated three-dimensional algorithm is highly attractive as it promises quick and accurate diagnosis, circumventing limitations associated with visual estimation or manual biplane measurements. We sought to assess the feasibility and correlation of such automated analysis with clinically established methods.

**Methods:**

A total of 198 patients undergoing transthoracic echocardiography (TTE) with assessment of left ventricular parameters by automated software algorithm (Philips 3D-Heartmodel; 3D-HM) which additionally had either left ventricular angiography (LVA) or cardiac magnetic resonance (CMR) within 24 h of the TTE examination were analyzed. Left ventricular parameters (left ventricular end-diastolic volume, LVEDV, left ventricular end-systolic volume, LVESV as well as left ventricular ejection fraction, LVEF) were compared between 3D-HM, CMR and LVA.

**Results:**

Correlation of left ventricular measurements was overall good to excellent and stronger for CMR (EF *r* = 0.824) than for LVA (EF *r* = 0.746). Unexperienced and expert clinicians yielded comparable good results. For CMR, highest correlation was detected in patients with BMI < 25 and excellent image quality. High agreement was seen between 3D-HM and CMR or LVA when stratifying patients according to heart failure categories.

**Conclusions:**

Echocardiographic quantification of left ventricular parameters using a software-based algorithm correlated well with established invasive and non-invasive modalities in the clinical setting, even for unexperienced clinicians. Such automated approaches are promising as they allow a reliable, more observer-independent as well as reproducible assessment of left ventricular function**.**

**Supplementary Information:**

The online version contains supplementary material available at 10.1007/s12574-022-00590-9.

## Introduction

Evaluation of left ventricular function by transthoracic echocardiography (TTE) is at the corner of many diagnostic and therapeutic decisions in cardiovascular care and one of the most often performed examinations in clinical practice. Measurement of left ventricular ejection fraction (LVEF) is often done by visual estimation from experienced echocardiographers or manual biplane measurement according to the Simpson method [1, 2]. Assessment of left ventricular parameters with three-dimensional echocardiography circumvents the limitations associated with two-dimensional echocardiography such as apical foreshortening and geometric assumptions and has been shown to be reliable and reproducible [3–5]. Use of three-dimensional echocardiography is therefore recommended by recent guidelines for the assessment of left ventricular volume parameters [2]. However, translation of this recommendation has been hampered by lack of experience of clinicians with three-dimensional echocardiography and the need for time-consuming three-dimensional reconstructions, limiting effective clinical workflow. Additionally, manually reconstructed 3D volumes might show substantial variability [6]. Novel, algorithm-based approaches are tempting as they possibly allow quick and accurate measurements for even unexperienced investigators. Yet, widespread use of such algorithms is limited due to uncertainty whether automated measurement of left ventricular function is reliable in daily practice. The aim of this work was therefore to compare left ventricular functional parameters obtained from three-dimensional transthoracic echocardiography using an automated software algorithm (HeartModel^A.I.^, Philips Healthcare) against qualitative, visual grading using left ventricular angiography and quantitative assessment of LV-function using cardiovascular magnetic resonance imaging (CMR), which is currently accepted as the clinical gold standard in this matter [7, 8].

## Methods

### Study population

**Fig. 1:**
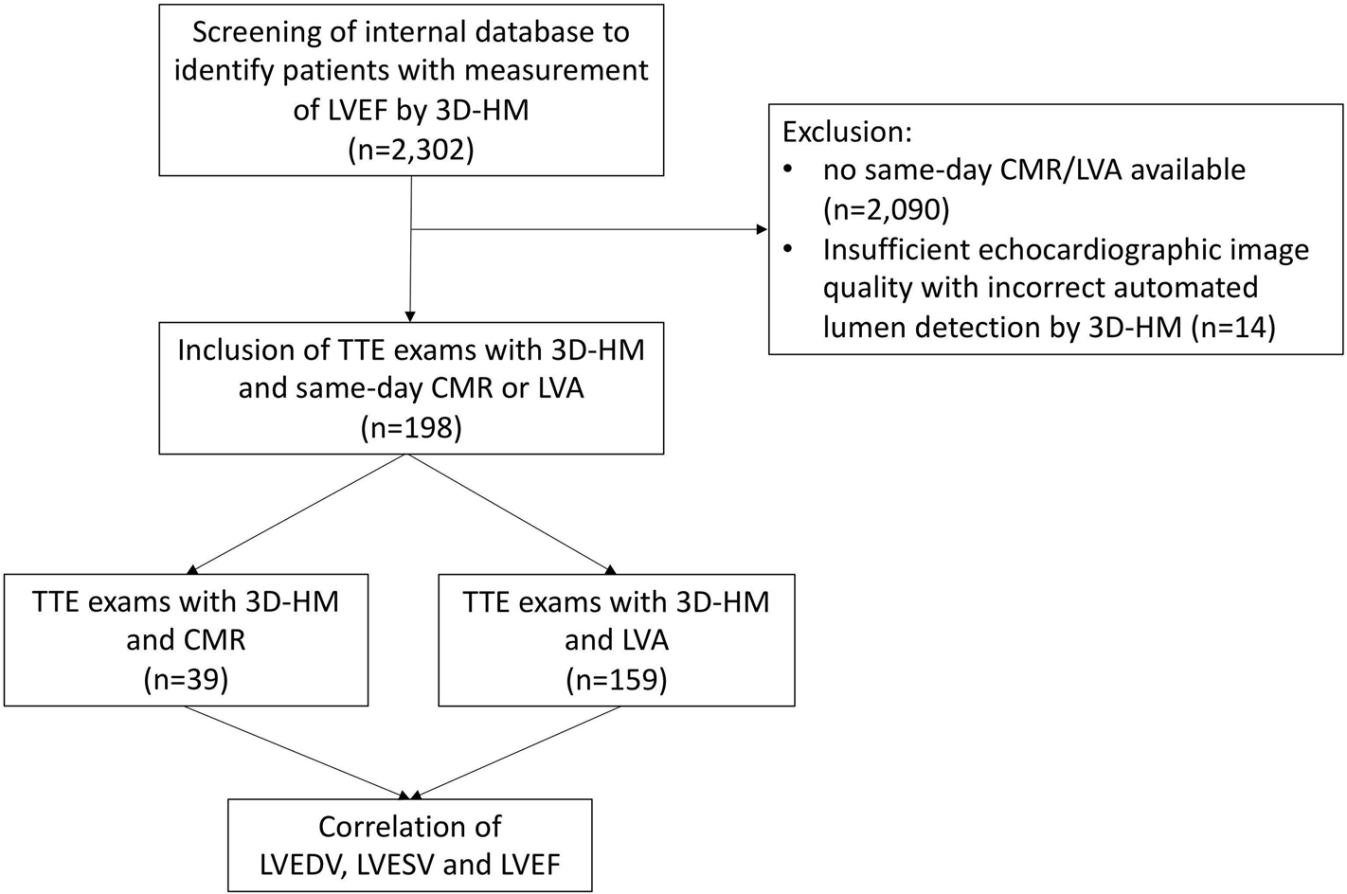
Study flowchart

For study flow, please see Fig. 1. Patients who received an AI-based analysis of left ventricular parameters (3D-HeartModel, Philips Medical Systems, Andover, MA, USA) between 07/2019 and 12/2020 were identified through screening of a large internal echocardiography database (*n* = 2302). In this population, patients who underwent either cardiac magnetic resonance (CMR) or invasive left ventricular angiography (LVA) within 24 h of the TTE examination were included in our analysis. Retrospectively, echocardiographic image quality was graded with respect to the visual tracing of the endocardial borders of the left ventricle and scored as “poor” if no endocardial border was visible, “sufficient” if the endocardial border was partially visible and “excellent” if the entire endocardial border was visible. All examinations graded as “poor” or in which automated lumen contour detection by 3D-Heartmodel was insufficient were excluded from the analysis (*n* = 14). Representative examples for each image quality category are shown in Supplementary Figure 5. A total of 198 examinations were available for analysis. For baseline characteristics of patients, please see Table 1.

**Table 1:** Baseline data

All	198 (100.0)
Age (years)	65.3 (1.7)
Female	63/198 (31.8)
Hypertension	139/198 (70.2)
Hypercholesterolemia	119/198 (60.1)
Diabetes mellitus II	31/198 (15.7)
Family disposition	62/198 (31.3)
Smoking	77/198 (38.9)
Body mass index (BMI)	26.3 (4.2)
Coronary artery disease (CAD)	
No CAD	67/198 (33.8)
1 V-CAD	42/198 (21.2)
2 V-CAD	39/198 (19.7)
3 V-CAD	50/198 (25.3)
Cardiomyopathy (CM)	
ICM	15/198 (7.6)
DCM	12/198 (6.1)
HCM	3/198 (1.5)
Other	4/198 (2.0)
No CM	164/198 (82.8)
NYHA class	1.15 (1.2)

Data are *n*/*N* (%) or mean (± SD)

### Transthoracic echocardiography

All 198 patients underwent standard transthoracic echocardiography using the EPIQ systems (Philips Medical Systems, Andover, MA, USA). The examination involved an apical four-chamber view which was acquired for initializing the 3D-HeartModel, allowing automated measurement of left ventricular end-diastolic volume (LVEDV), left ventricular end-systolic volume (LVESV) as well as left ventricular ejection fraction (LVEF). Manual correction of automated lumen contour detection by 3D-Heartmodel was done at the discretion of the echocardiographer. All measurements, images and loops were stored offline. Observer variability of the 3D-Heartmodel was measured by the same clinician within 24 h of the examination (intraobserver variability) and by two blinded clinicians within the same examination (interobserver variability). Clinicians performing the examination were classified based on the level of experience as “beginner” (< 6 months experience in performing standard TTE) or “expert” (>6 months experience).

### Cardiac magnetic resonance

Cardiac magnetic resonance imaging was performed in conformity with the recommendations on standardized CMR imaging in 39 subjects and indicated for the assessment of ischemic and nonischemic cardiomyopathies. All subjects underwent a CMR examination in a 1,5-Tesla MRI scanner (Magnetom Avanto, Siemens Healthcare, Erlangen, Germany) using cardiac‐dedicated phased‐array coils. Continuous monitoring of vital signs throughout the entire CMR scan was performed with a 4-lead ECG. The CMR protocol comprised of functional imaging, T2 imaging, and assessment of relative and late gadolinium enhancement (LGE). Functional imaging was performed using steady‐state free‐precession cine sequences in short axis (SA), horizontal long axis (HLA), vertical long axis, and left ventricular outflow tract orientation. Fat‐suppressed T2‐weighted black‐blood turbo spin echo images in SA and HLA were obtained and allowed for visualization of myocardial edema. The following imaging parameters were used: field of view 340 × 340 mm; acquisition matrix 256 × 256; reconstruction matrix 512 × 512; slice thickness was 6–8 mm, with 2–4 mm interslice gaps; spatial resolution 0.65 × 0.65 × 0.65 mm, etc.

### Quantitative CMR image analysis

CMR datasets were transferred to a dedicated workstation and functional analysis was performed on a commercially available software program (CVI 42, Version 5.6, Circle Cardiovascular Imaging Inc., Canada). The end-diastolic and end-systolic phases were determined for each CMR dataset. For the detection of each phase, the largest and narrowest diameters of the ventricular cavity at the middle of the ventricle were used. The endocardial and epicardial borders were traced manually in short axis images in both phases. The basal segment of the left ventricle was defined as the most basal slice that was surrounded by at least 50% of the myocardium filled with blood and was included in the LV chamber volume. The apex was defined as the last slice with a visible lumen throughout the entire cardiac cycle. The ESV, EDV, SV, EF, and LV myocardial masses were determined according to the Simpson’s rule.

### Left ventricular angiography

Left ventricular angiography was performed in 159 patients during left heart catheterization according to current standard of care using commercially available contrast dye (Imeron^®^ 350, Bracco Imaging Deutschland GmbH) and simultaneous X-ray image acquisition. Ejection fraction was graded visually by an expert clinician.

### Subanalysis

To investigate factors associated with higher or lower correlation, cases were further divided into three subgroups: (1) experience of the echo-performing clinician (“beginner” versus “expert”), (2) echocardiographic imaging quality (“good” versus “excellent” image quality) and (3) body mass index (BMI, <25 versus >25).

### Statistical analysis

Data are presented as mean ± standard deviation. Correlation and agreement between measurement of LVEDV, LVESV and LVEFVF were assessed by two-sided Pearson’s correlations and Bland–Altman comparisons. Intra- and interobserver variability was measured using intraclass correlation coefficients (ICC). All statistical analyses were performed using commercially available software (SPSS, Version 28, IBM Corporation, Armonk, NY). A *p* value of < 0.05 was considered statistically significant.

## Results

In total, transthoracic studies from 198 patients were suitable for analysis.

Within this cohort, 39 (19.7%) patients received cardiac magnetic resonance imaging (CMR) and 159 (80.3%) left ventricular angiography within 24 h to the echocardiographic examination. Echocardiographic image quality was graded as “poor” in *n* = 0 patients (0%), “good” in *n* = 75 patients (37.9%) and “excellent” in *n* = 123 patients (62.1%). Left ventricular parameters such as LVEF, LVEDV and LVESV as derived from CMR and 3D-heart model were 53.1% (± 11.5), 160.3 ml (± 51.4), 78.3 ml (± 46.9) and 53.9% (± 14.9), 167.1 ml (± 65.5) and 82.9 ml (± 60.5) respectively, showing no significant differences between the two aforementioned methods (all *p* > 0.05, see Table 2). In the group of patients that underwent left ventricular angiography and echocardiography (*n* = 159), LVEF, LVEDV and LVESV derived from 3D-Heartmodel were 55.1 % (± 9.5), 164.8 ml (± 76.0) and 78.0 ml (± 55.2), respectively. Comparison of left ventricular angiography and 3D-Heartmodel showed no significant difference with respect to left ventricular ejection fraction (*p* > 0.05, see Table 2).

**Table 2:** Left ventricular parameters in patients with 3D-HM and CMR (*n* = 39) and 3D-HM and LVA (*n* = 159)

	EF (%)	EDV (ml)	ESV (ml)
3D-HM (*n* = 39)	53.1 (11.5)	160.3 (51.4)	78.3 (46.9)
CMR (*n* = 39)	53.9 (14.9)	167.1 (65.5)	82.9 (60.5)
*p*	*0.405*	*0.648*	*0.936*
3D-HM (*n* = 159)	55.1 (9.5)	164.8 (76.0)	78.0 (55.2)
LVA (*n* = 159)	54.2 (9.0)	n.a	n.a
*p*	*0.437*	*n.a*	*n.a*

Data are mean (± SD), a p value of > 0.05 was considered statistically significant

Correlation of left ventricular parameters was assessed using Pearson’s method. Correlation of LVEF for measurement by 3D-Heartmodel and cardiac magnetic resonance was overall good to excellent with *r* = 0.824, *p* < 0.0001, while correlation of LVEDV and LVESV was *r* = 0.921 and *r* = 0.944, respectively.

**Fig. 2:**
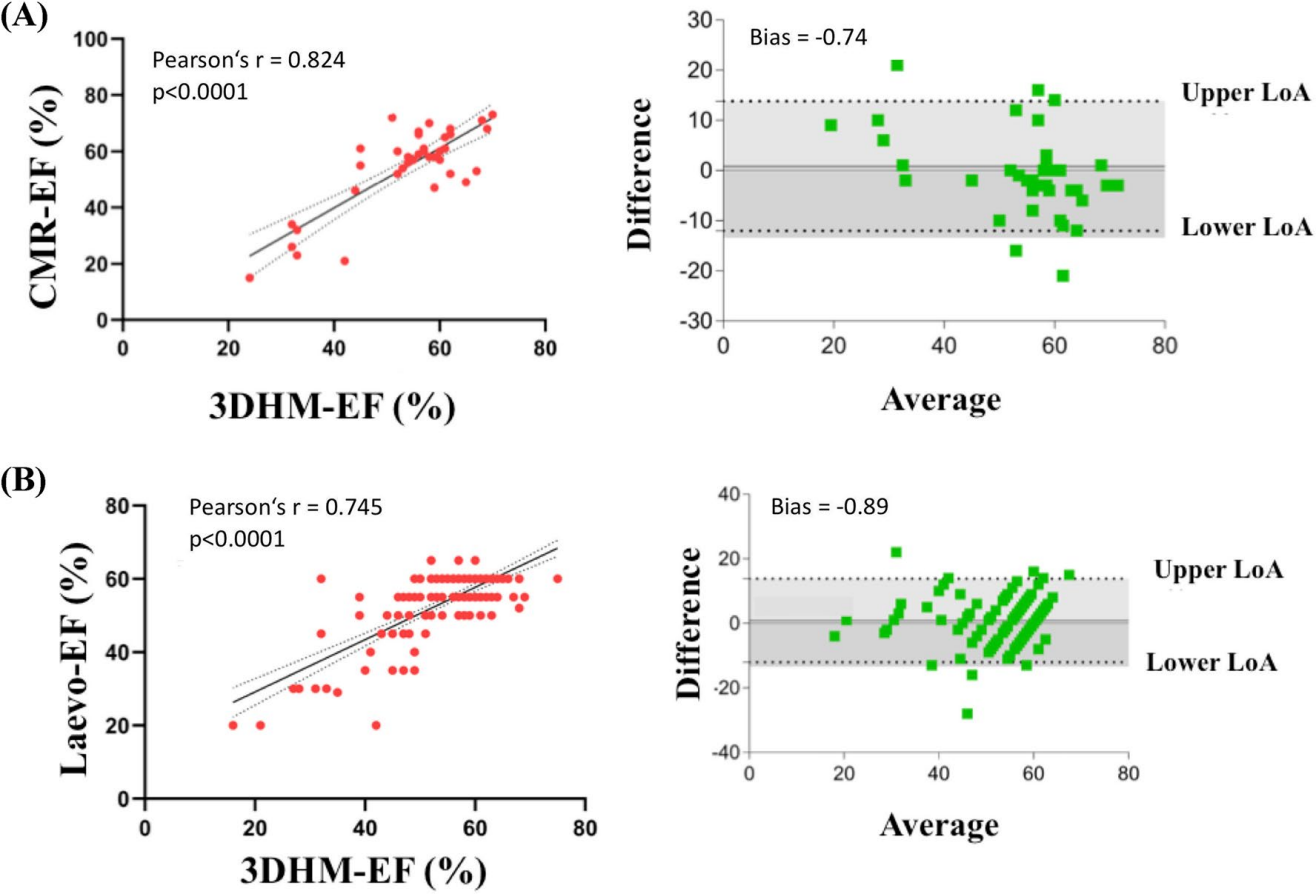
Correlation (left) and Bland–Altman plots (right) for left ventricular ejection fractions comparison. **A** 3D-HM versus CMR **B** 3D-HM versus left ventricular angiography. Correlation plots: the solid line is the line of identity, while the dashed line is the regression line. Abbreviations: 3D-HM, Echocardiography-based 3D-heart model; *CMR* cardiovascular magnetic resonance imaging

Correlation of 3D-Heartmodel versus left ventricular angiography was less pronounced, however still good with *r* = 0.745, *p* < 0.0001. Similar, Bland–Altman plots demonstrated good agreement for both modalities with a bias of − 0.74 for CMR and − 0.89 for LVA. (see Table 3 and Fig. 2). Intra- and interobserver variability of measuring LVEF using 3D-Heartmodel was low (ICC = 0.979 and 0.917, respectively). When comparing examinations based upon experience of the clinician, there was overall high significant correlation between 3D-HM and CMR in clinicians with low experience (beginner, EF *r* = 0.815, *p* = 0.002) and expert echocardiographers (EF *r* = 0.838, all *p* < 0.001). Comparing 3D-HM and LVA yielded similar results, however with lower correlation with EF *r* = 0.605 and *r* = 0.765, respectively (all *p* < 0.05, see Table 4)

**Table 3:** Pearson’s correlation (*r*) for 3D-HM versus CMR and 3D-HM versus LVA in all cases (*n* = 198)

	*r*	*p*
3D-HM vs. CMR		
EF (%)	0.824	< 0.0001*
EDV (ml)	0.921	< 0.0001*
ESV (ml)	0.944	< 0.0001*
3D-HM vs. LVA		
EF (%)	0.745	< 0.0001*

*Statistically significant correlation (p<0.05)

**Table 4:** Pearson’s correlation (*r*) for 3D-HM versus CMR and 3D-HM versus LVA in different stages of clinical experience (Beginner *n* = 49 and Expert *n* = 149)

	Beginner		Expert	
	*r*	*p*	*r*	*p*
3D-HM vs. CMR				
EF (%)	0.815	0.002*	0.838	< 0.001*
EDV (ml)	0.856	0.14*	0.952	< 0.001*
ESV (ml)	0.970	< 0.001*	0.957	< 0.001*
3D-HM vs. LVA				
EF (%)	0.605	< 0.001*	0.765	< 0.001*

*Statistically significant correlation (p<0.05)

**Fig. 3:**
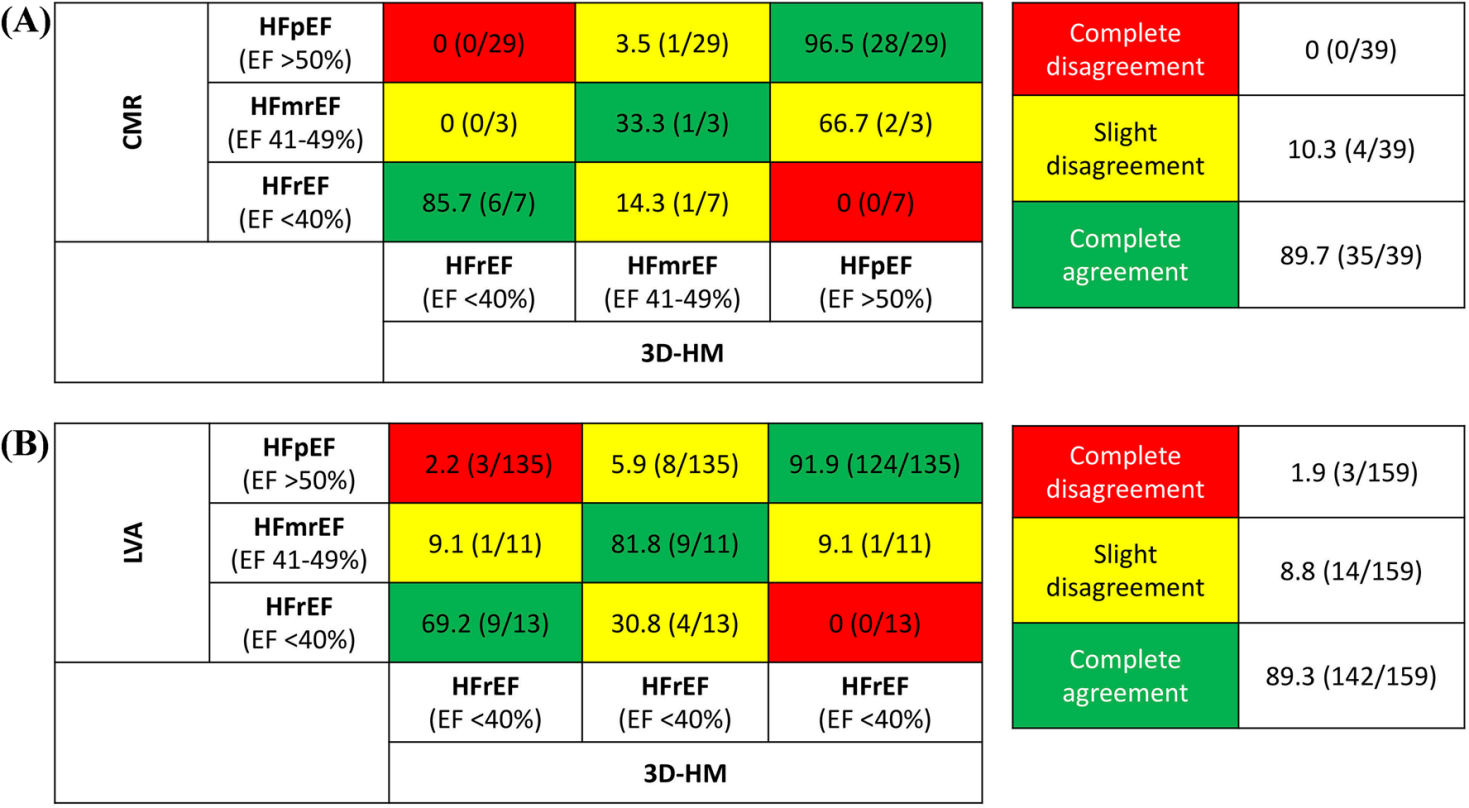
Stratification of heart failure categories with 3D-HM. Agreement regarding the classification of heart failure categories between **A** CMR and 3D-HM and **B** LVA and 3D-HM. Green color indicates correct classification while yellow color indicates slight disagreement ± 1 class between CMR/LVA and 3D-HM (e.g., HFpEF classified as HFmrEF). Red color indicates complete disagreement ± 2 classes (e.g., HFpEF classified as HFrEF). Data are % (*n*/*N*)

Next, we aimed to understand whether 3D-HM could confirm the determination of heart failure class by CMR or LVA: all patients were categorized into (1) EF < 40% (= heart failure with reduced EF or HFrEF), (2) EF 41-49% (= heart failure with mildly reduced EF or HFmrEF) or (3) EF > 50% (= heart failure with preserved EF or HFpEF). High agreement was seen between CMR and 3D-HM as well as LVA and 3D-HM regarding confirmation of heart failure class (Fig. 3): this means that for the majority of patients, respective heart failure category (determined by CMR) was confirmed when using 3D-HM (agreement of 85.7% of cases for HFrEF, 33.3% for HFmrEF and 96.5% for HFpEF, Fig. 3a). Similarly, when comparing LVA with 3D-HM, agreement was seen for 69.2% in cases of HFrEF, 81.8% for HFmrEF and 91.9% for HFpEF (Fig. 3b). In total, complete agreement was detected in 89.7% of patients (CMR with 3D-HM) and 89.3% (LVA with 3D-HM).

Finally, we aimed to investigate the impact of echocardiographic image quality upon correlation of 3D-Heartmodel with CMR and LVA: in echocardiographic examinations with image quality graded as “good”, LVEF measurement by 3D-Heartmodel and cardiac magnetic resonance was *r* = 0.806, *r* = 0.874 for EDV and *r* = 0.945 for ESV. *R* for correlation of 3D-Heartmodel versus left ventricular angiography was 0.694. In patients in which image quality was scored as “excellent”, correlation was slightly higher for EF and EDV (*r* = 0.827 and *r* = 0.919) and slightly lower for ESV (*r* = 0.940, see Table 5). Additionally, patients with an obese phenotype (BMI >25) showed lower correlation between 3D-Heartmodel and cardiac magnetic resonance for EF (*r* = 0.720 in patients with BMI >25 versus *r* = 0.889 for patients with BMI < 25) and EDV (*r* = 0.891 versus *r* = 0.940) but similar correlation for ESV (0.959 in patient with BMI < 25 and 0.917 in BMI > 25, see Table 6).

**Table 5:** Pearson’s correlation (*r*) for 3D-HM versus CMR and 3D-HM versus LVA in “good” and “excellent” image quality (*n* = 75 and *n* = 123)

	Image quality “good”		Image quality “excellent”	
	*r*	*p*	*r*	*p*
3D-HM vs. CMR				
EF (%)	0.806	< 0.0001*	0.827	< 0.0001*
EDV (ml)	0.874	< 0.0001*	0.919	< 0.0001*
ESV (ml)	0.945	< 0.0001*	0.940	< 0.0001*
3D-HM vs. LVA				
EF (%)	0.694	0.001*	0.776	0.001*

*Statistically significant correlation (p<0.05)

**Table 6:** Pearson’s correlation (*r*) for 3D-HM versus CMR and 3D-HM versus LVA in BMI < 25 and BMI > 25 (*n* = 82 and *n* = 111, no BMI available for *n* = 5)

	BMI < 25		BMI > 25	
	*r*	*p*	*r*	*p*
3D-HM vs. CMR				
EF (%)	0.889	0.001*	0.720	0.001*
EDV (ml)	0.940	< 0.0001*	0.891	< 0.0001*
ESV (ml)	0.959	< 0.0001*	0.917	< 0.0001*
3D-HM vs. LVA				
EF (%)	0.596	0.001*	0.811	< 0.0001*

*Statistically significant correlation (p<0.05)

## Discussion

The aim of this study was to compare parameters of left ventricular function (i.e., LVEF, LVEDV and LVESV) derived by a novel, algorithm-based analysis (3D-Heartmodel^A.I.^) with clinically established quantification (i.e., same-day measurement done by either cardiac magnetic resonance or left ventricular angiography). The main findings of our work are given as follows:

1. Measurement of left ventricular parameters with 3D-Heartmodel showed strong correlation with analysis regarded as gold standard
2. Correlation was stronger for biplane measurement using cardiac magnetic resonance than for visual estimation with left ventricular angiography.
3. Clinical experience did not have an impact upon correlation, supporting the use of 3D-HM even for unexperienced clinicians.
4. Using 3D-Heartmodel, approximately 90% of patients were correctly stratified into heart failure class (HFpEF, HFmrEF and HFrEF)
5. Highest correlation of LVEF and LVEDV was seen in patients with normal BMI and excellent image quality.

Evaluation of left ventricular function is extremely important in daily clinical care. Accurate and reliable evaluation of left ventricular ejection fraction in the clinical setting is essential, since it has a major impact on further diagnostic testing and clinical workflow and is most frequently performed by two-dimensional transthoracic echocardiography with Simpsons’ biplane measurements. This is especially true for heart failure patients where adequate monitoring of disease progression and pharmacological treatment is essential [9, 10]. However, limitations associated with two-dimensional echocardiography, such as foreshortening or poor endocardial demarcation especially in patients with unfavorable characteristics, make the use of biplane measurements of left ventricular parameters error-prone and observer-dependent [11, 12]. We speculate that the use of an automated algorithm may help to reduce time-consuming collection of left ventricular data, which is beneficial in high-volume echocardiography laboratories. As such, we were able to demonstrate that the proposed method yielded satisfying results even in patients with obese phenotype with BMI > 25 (*r* = 0.720).

**Fig. 4:**
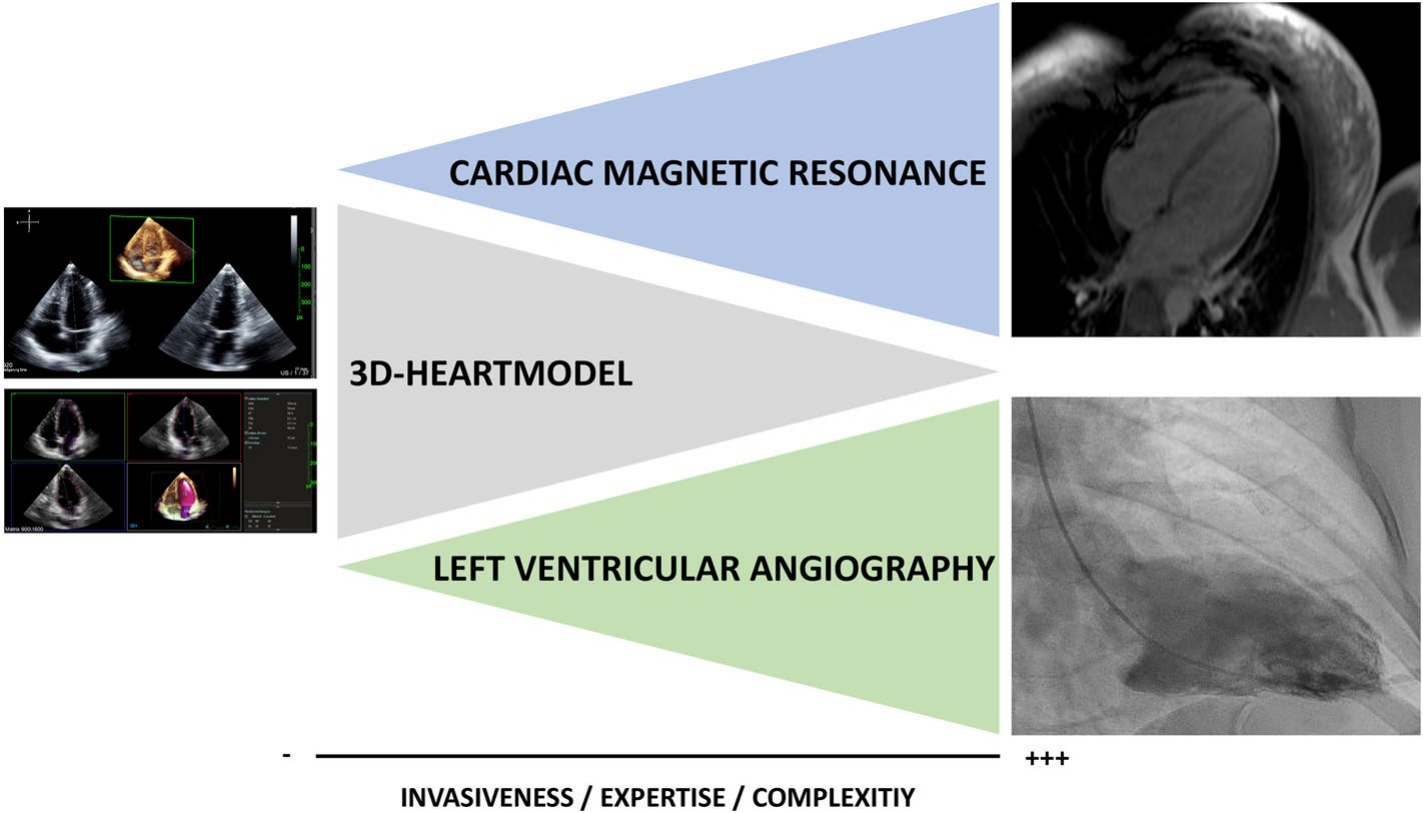
Comparison of 3D-Heartmodel, CMR and LVA

Semi-quantitative assessment of left ventricular dimensions using cardiac magnetic resonance is considered the gold standard for quantification of left ventricular dimensions [8] but local availability of CMR is often limited, requires expert radiologists and is contraindicated in a significant subset of patients (e.g., after implantation of a pacemaker or ICD). Likewise, left ventricular angiography allows detailed visualization of the left ventricle [13]. However, this technique has several limitations since its monoplane technique is often inaccurate, its application is limited due to its invasiveness and the need for contrast medium and radiation as well as possible side effects associated with catheterization of the left ventricle. Echocardiography on the other side is widely available, non-invasive, inexpensive and free of radiation (Fig. 4). Three-dimensional echocardiography could circumvent the limitations associated with two-dimensional echocardiography [10]. As compared with cardiac magnetic resonance, three-dimensional echocardiography has been shown to be more accurate and precise than conventional two-dimensional measurement but underestimating left ventricular volumes [6], being cumbersome, time-consuming and requiring special expertise. Therefore, clinical application has still been limited. Recently developed novel software-based tools with automated and quick 3D-rendering (such as the 3D-Heartmodel) use a distinct algorithm trained upon approximately thousand different echocardiographic imaging data sets enabling automated and quick contour detection of the left ventricle in end-diastole and end-systole [14] and was shown to be accurate and more reproducible in estimating left ventricular volumes compared with manual 3D-echocardiography [15, 16]. This allows user-friendly and more investigator-independent measurement of left ventricular parameters. Several studies have shown that inter- and intraobserver variability of 3D-Heartmodel is fairly low, allowing high reproducibility of this method in clinical routine [14, 17, 18]. In this study, we decided to retrospectively investigate the real-world performance of 3D-Heartmodel, comparing it with two distinct approaches often used in daily practice: on the one hand, we used same-day magnetic resonance imaging with multiplane quantification of LVEDV, LVESV and derived LVEF by an expert radiologist. On the other hand, we used same-day left ventricular angiography obtained during left heart catheterization, in which LVEF was visually graded by an expert clinician.

Our results demonstrate a strong correlation of LVEF between 3D-Heartmodel and CMR (*r* = 0.824), which is in accordance with previous findings:

Recently, a comparison between 3D-Heartmodel and manual 3D echocardiography showed good correlation (*r* = 0.79) [19]. Additionally, a study of 65 patients undergoing 3D-echocardiography in combination with the first generation of the AI-based heart model algorithm and magnetic resonance imaging showed high agreement between these two modalities with respect to left ventricular parameters (*r* = 0.84–0.95) [14]. Similar, Levy at al. found an equally high and significant correlation of LVEF between 3D-Heartmodel and CMR with *r* = 0.93 [17] which was later confirmed in a study of 20 patients by Narang et al. [20]. Finally, comparison of 3D-Heartmodel showed good agreement with conventional biplane method, manual 3D-echocardiography and CMR in 200 patients [21]. However, such studies mainly using experienced sonographers for echocardiographic imaging in a preset setting may not reflect real-life conditions where often unexperienced sonographers are involved. Therefore, our retrospective analysis is informative as the echocardiographic examinations done in our study were performed by clinicians with varying expertise in echocardiography, demonstrating high correlation even in unexperienced clinicians.

Additionally, we demonstrate that the 3D-Heartmodel is able to stratify the overwhelming majority of patients into correct heart failure categories (approximately 90% for both CMR and LVA). Hence, even though the EF might be slightly under- or overestimated, 3D-HM could allow proper risk stratification of patients with heart failure, which is important for therapeutic decision such as intensified pharmacotherapy.

To the best of our knowledge, no study has so far investigated the comparison of 3D-Heartmodel with visual estimation of left ventricular function by left ventricular angiography. While left ventricular angiography is no longer regarded as the clinical gold standard for quantification of left ventricular function and visual estimation of left ventricular function can be criticized for its possible inaccuracy, it is the authors’ experience that it is still widely used in daily clinical practice.

In conclusion, an echocardiographic-based automated software algorithm to obtain left ventricular functional parameter showed excellent agreement with clinically established methods such as CMR or invasive angiography and can easily be used in the clinical setting. Our findings support the use and application in daily clinical practice. Larger, prospective studies are needed to validate our findings and to assess its impact of subsequent clinical work-up.

## Supplementary Information

Below is the link to the electronic supplementary material.

Supplementary file1 (TIFF 831 kb) Representative echocardiography samples with different levels of image quality (A) Excellent image quality: Delineation of endocardial borders is fully possible (B) Good image quality: Delineation of endocardial borders is mostly possible excepts small segments (such as the apex). (C) Poor image quality: Delineation of endocardial borders is feasible in less than 50%

## Data Availability

The data that support the findings of this study are available from the corresponding author upon reasonable request.
